# Reactive Case Detection (RACD) and foci investigation strategies in malaria control and elimination: a review

**DOI:** 10.1186/s12936-020-03478-0

**Published:** 2020-11-10

**Authors:** Ruwanthi Perera, Amandhi Caldera, A. Rajitha Wickremasinghe

**Affiliations:** Department of Public Health, Faculty of Medicine, University of Kelaniya, P. O. Box 6, Thalagolla Road, Ragama, 11010 Sri Lanka

**Keywords:** Reactive case detection, Malaria elimination, 1-3-7 strategy, Malaria control, Foci investigation

## Abstract

**Background:**

Reactive case detection (RACD) and foci investigation are key strategies in malaria elimination and prevention of its re-establishment. They are a key part of surveillance that has been recommended by the World Health Organization (WHO) to be considered as a core intervention and as one of the three pillars of the Global Technical Strategy for Malaria 2016–2030.

**Methods:**

A search using the key words “Reactive Case Detection”, “RACD”, “RCD” and “Malaria” was carried out in PubMed, Scopus, Taylor and Francis online databases for studies published until 31st July 2019. The inclusion criteria for selection of articles for review included (1) how RACD is implemented in each country; (2) challenges faced in RACD implementation; (3) suggestions on how the effectiveness of RACD process can be improved.

**Results:**

411 titles were identified, 41 full text articles were screened and 29 were found eligible for inclusion in the review. Published literature on RACD, and case and foci investigations has mostly assessed the process of the activity. Most studies have documented that the yield of positives in RACD has been highest in the index case’s household and the immediate neighbourhood of the index case. Microscopy and RDTs are the common tests used in RACD. The guidelines for case and foci investigation, and RACD and PACD, are not universally adopted and are country-specific. Some of the limitations and challenges identified include lack of proper guidelines, logistic issues and problems with public compliance.

**Conclusions:**

Although there is no documented evidence that RACD is useful in malaria elimination settings, most authors have opined that RACD is necessary for malaria elimination. Lack of knowledge in the target populations, a target radius and how to carry out the RACD process is a major challenge in the decision-making process.

## Background

Renewed global interest in malaria elimination since the beginning of this Century has motivated countries to plan for and implement evidence-based strategies moving from malaria control to elimination. The Global Technical Strategy for Malaria 2016–2030 of the World Health Organization (WHO) has made transforming surveillance into a core intervention as one of its three pillars [1]. Surveillance is “the continuous and systematic collection, analysis and interpretation of disease-specific data, and the use of that data in the planning, implementation and evaluation of public health practice” [2]. In malaria elimination settings, surveillance includes case and foci investigation and classification to provide information for response to identify all infections and to prevent onward transmission. Part of case and foci investigation involves active case detection when an index case is reported.

Active Case Detection (ACD) is defined as the detection by health workers of malaria infections at community and household level in population groups that are considered to be at high risk. ACD may be conducted as fever screening followed by parasitological examination of all febrile patients or as parasitological examination of the target population without prior screening [3]. Active case detection is used to fill gaps in the passive case detection system and to detect malaria infections as early as possible in populations that are at high risk of infection [3]. ACD in malaria elimination settings can be of two types: (1) Proactive Case Detection (PACD), and (2) Reactive Case Detection (RACD or RCD).

Proactive case detection involves screening high risk populations without the trigger of a passively detected case [4]. RACD, originally referred to as ‘contact tracing’ during the Global Malaria Eradication programme [5], is the process of identifying further cases following the detection of a case; it may involve testing of co-travellers and co-workers who may have experienced the same exposure or household members and neighbours resident in the vicinity of the index case [6]. Countries engage in a wide variety of activities that they consider to fall within the scope of their ACD strategy, and may include PACD and/or RACD [4, 7].

RACD is triggered by passively detected cases and involves screening households or individuals within a specified area, typically within a pre-determined radius, around a locally acquired or imported case, with the goal of preventing further malaria transmission by identifying additional infections, symptomatic or asymptomatic [4, 6, 7]. RACD which includes screening of communities for malaria around an index case followed by immediate treatment of positive cases, usually coupled with vector control interventions and health education, has been or is widely adopted with varying methodologies in many countries to achieve and maintain malaria elimination, despite little evidence to support the impact of RACD on transmission ([4, 8–17].

Unlike passive case detection (PCD), RACD is a potentially effective strategy for detecting afebrile malaria infections which tend to cluster in low-transmission settings [18]. The ability of RACD to detect afebrile infections depends on the sensitivity of the diagnostic test being used. It is likely that standard field diagnostic methods [Rapid Diagnostic Tests (RDTs) and microscopy] will fail to detect a substantial proportion of low-density parasitaemic infections [11, 13–16, 18–20].

This systematic review was conducted to appraise the different approaches countries follow in conducting RACD, foci investigation, factors affecting RACD, tests used, and the challenges they face.

## Methods

### Search strategy

A search using the key words “Reactive Case Detection”, “RACD”, “RCD” and “Malaria” was carried out in PubMed, Scopus, Taylor and Francis online databases for studies published until 31st July 2019. To reduce publication bias, grey literature were explored using Google scholar and Paperity using the phrases “malaria” AND “reactive case detection” AND “greater mekong sub region”, “malaria” AND “reactive case detection” AND “1-3-7”, “malaria” AND “reactive case detection” AND “1-3-7” AND “elimination”.

One author screened the search results to exclude books, conference abstracts/posters and papers that were clearly irrelevant and non-English articles. The remaining papers were evaluated for full text review if they contained information on the implementation of RACD either as an evaluation of the process or as an experimental approach, and reviewed, if selected. The inclusion criteria for selection of articles for review included: (1) how RACD is implemented in each country; (2) challenges faced in RACD implementation; (3) suggestions on how the effectiveness of RACD process can be improved.

## Results and discussion

**Fig. 1:**
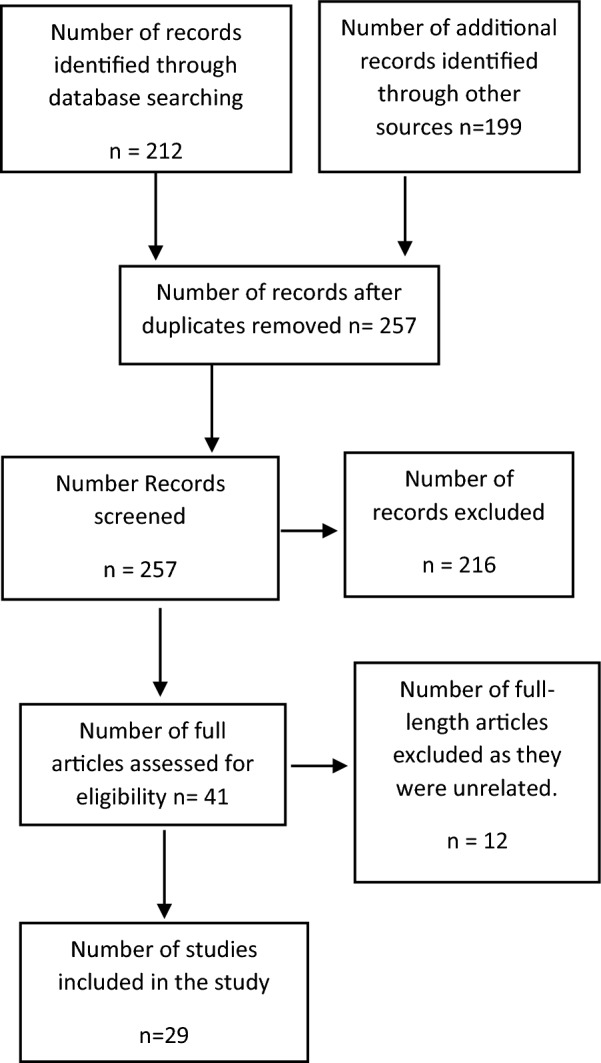
Article selection strategy

Figure 1 shows the number of articles found and later kept or excluded during screening. 411 titles were identified, which were reduced to 257 after removal of duplicates; 216 titles were either not full-text articles, or were non-English, or were not related to RACD, and were excluded. The remaining 41 full text articles were screened and 29 were found eligible for inclusion in the review.

Of the 29 studies reviewed, 10 were done in the Greater Mekong Subregion (GMS); two were done in Latin America and the rest in Africa. Among countries in the GMS, 5 studies were related to the 1-3-7 strategy followed by China, one study was related to the RACD techniques in Myanmar, 3 studies were from Cambodia, and one study was from Viet Nam (Table 1).

**Table 1:** List of studies included in the review

Citation	Journal	Source	Country/region covered	Comments
Aidoo et al. [25]	Malaria Journal	PubMed	Kenya	
Bansil et al. [30]	Malaria Journal	PubMed	Ethiopia	
Cao et al. [8]	PLoS Medicine	Paperity	China	1-3-7
Cotter et al. [31]	Malaria Journal	PubMed, Scopus, Google scholar	China	1-3-7
Fontoura et al. [33]	PLOS Neglected Tropical Diseases	PubMed	Brazil	
Gerardin et al. [39]	Malaria Journal	PubMed, Google Scholar	Zambia	Recommended for settings where transmission has recently been reduced
Hsiang et al. [10]	Clinical Infectious Diseases	PubMed	Eswatini	
Hustedt et al. [29]	Malaria Journal	PubMed, Scopus, Paperity	Cambodia	Follow up cases within 3 days of detection
Kyaw et al. [48]	Tropical Medicine and Health	Google Scholar, Paperity	Myanmar	Malaria Notification, Investigation, Classification and Response strategy (similar to 1-3-7)
Larsen et al. [47]	Malaria Journal	PubMed, Paperity	Zambia	
Larsen et al. [37]	Malaria Journal	PubMed, Paperity	Zambia	location as the most important parameter in RACD
Larson et al. [26]	Malaria Journal	PubMed, Paperity	Zambia	
Littrell et al. [11]	Malaria Journal	PubMed, Paperity	Senegal	Notified within 24 h and investigated within 3 days
Lu et al. [45]	Infectious Diseases of Poverty	Google Scholar	China	1-3-7
Lolina Gómez et al. [27]	PLOS Neglected Tropical Diseases	PubMed	Colombia	
Parker et al. [38]	Malaria Journal	Paperity	Myanmar, Myanmar-Thailand border	
Pinchoff et al. [35]	Malaria Journal	PubMed	Zambia	Spatial patterns
Rossi, Van den Bergh et al. [28]	Clinical Infectious Diseases	PubMed, Scopus, Oxford Journals, Google Scholar	Cambodia	Screening household members
Searle et al. [13]	Malaria Journal	PubMed, Paperity	Zambia	Reactive screen-and-treat programme
Smith Gueye et al. [4]	Malaria Journal	PubMed	China	
Sturrock et al. [15]	PLoS ONE	PubMed, Paperity	Swaziland	Screening radius
Tambo et al. 2018 [43]	Malaria Journal	PubMed	Namibia	RDT Vs LAMP in RACD
van Eijk et al. [9]	Malaria Journal	PubMed	India	RCD is low malaria transmission areas
Wang et al. [52]	Malaria Journal	PubMed, Scopus, Google Scholar	China-Myanmar border-included 4 countries	1-3-7
Xiao et al. [24]	American Journal of Tropical Medicine and Hygiene	Google Scholar	China-Myanmar border 18 counties	1-3-7
Yukich et al. [53]	Malaria Journal	PubMed	Zambia	RCD, fDA
Zelman et al. [49]	Malaria Journal	PubMed	Indonesia	
Zemene et al. [32]	Malaria Journal	PubMed, Paperity	Ethiopia	
Zhou et al. [22]	Infectious Diseases of Poverty	PubMed, Scopus, Google Scholar	China	1-3-7

### 1-3-7 strategy in China and its adaptations

China launched its malaria elimination programme in July 2010 with a plan to achieve elimination by 2020 [21]. China’s 1-3-7 RACD strategy had time-bound targets for case reporting, investigation and foci response activities. The “1-3-7” refers, respectively, to reporting of malaria cases within 1 day using the web-based China Information System for Disease Control and Prevention (CISDCP); their confirmation by double reading of slides by expert microscopists, and, where possible, quality assured PCR at a provincial laboratory, and investigation and classification is completed within 3 days; and the appropriate public health response to prevent further transmission to be done within 7 days [22].

Focus investigation and action are carried out within 7 days irrespective of whether a case is classified as local or imported. The area around a case (the “focus”) is investigated to evaluate the risk of local transmission. Different actions, triggered based on the results of the investigation, are completed within 7 days. RACD is done based on the classification of a focus; in “inactive” (areas that do not support transmission due to the absence of vectors during the non-transmission season or without capable vectors) and “pseudo” (imported cases reported in a malaria-free area) foci, RACD screening is carried out in contacts of the case (‘‘hot populations’’), such as co-workers who travelled to the same area. Where viable vectors are identified and ecological conditions are suitable for malaria transmission, the focus is classified as an ‘‘active focus’’, and more intensive RACD (up to 200 neighbours) and vector control are initiated. RACD is conducted using RDTs for immediate results, with filter paper blood spots collected from all persons screened for later molecular (PCR) testing to detect low-density infections that may be missed by RDTs [9, 16, 20]. If local transmission is possible or confirmed, targeted action to seek out other infections and reduce the chance of onward transmission is completed within seven days [22]. The performance of the strategy has improved from the control to the elimination phase in terms of higher percentages of compliance to the “1-3-7” time lines [21, 22].

China’s 1-3-7 strategy or its variations have been adopted and adapted in many other countries [8, 23]. Along the China-Myanmar border, the approach was well executed except for the “3” indicator, which was 96% accomplished on average in the 18 border counties. While acknowledging the need for a well-planned and executed surveillance system for malaria elimination, given the few malaria cases RACD detected, the authors state that there is no evidence to suggest that it was effective, and if effective, the extent of its effectiveness [24].

In the Asia Pacific region, the practice of case investigation varies widely, the trigger typically being a single case report or a defined threshold of multiple cases [4]. It was reported that case investigation is part of surveillance activities where a broad array of demographic data is collected using different definitions for imported cases. The spatial range of screening varies from a specific number of households to an entire administrative unit (e.g., village) but the optimal radius is unclear [4]. The strategy is labour intensive and expensive; in addition, the common detection methods used, microscopy or a rapid diagnostic test, may miss low-density infections that are still capable of transmitting malaria infections [4].

### Factors associated with RACD positivity

#### Geographic extent

Many studies have reported different strategies with regard to the geographic extent of RACD [10, 25–27]. Some studies have included only household members [28], while others have extended the area to include residents in the immediate neighbourhood of index cases to distances varying from 140 metres to one kilometre [26–29]. Some studies have screened 50–200 persons [8, 10, 11, 30–32]. Most of the positive cases detected by RACD have been in close geographic proximity to the residence of the index cases. Cases detected further away tended to be genetically unrelated to the index infection [33]. None of the studies reviewed had reported on the target radius for RACD in urban settings, where population densities are higher requiring more persons to be screened for a given radius, further burdening the health system.

Several important associations with RACD positivity were reported in individual studies. One study in Zambia detected *Plasmodium falciparum* gametocytes using PCR in 2.4% (2/87) of index case household members and 0% (0/141) among other contacts (p = 0.145) [9]. In Swaziland (now Eswatini), significantly higher odds of malaria among contacts within the 1st week from presentation of the index case were reported compared to more than 2 weeks; the odds was reduced if the index house was sprayed [10]. In Thailand, PCR identified four cases which were missed by routine microscopy [34]. In Zambia that has a high prevalence of malaria, household contacts were significantly more likely to be positive when the index case was < 5 years, and with increasing distance from the main road [4, 35].

In the Western Cambodian province of Pailin, *P. falciparum* infection was associated with fever (p = 0.013), being a member of a control household (p < 0.001), having a history of malaria infection (p = 0.041), and sleeping without a mosquito net (p = 0.011) [29]. Significant predictors of *Plasmodium vivax* infection, diagnosed by PCR, were fever (p = 0.058) and history of malaria infection (p < 0.001) [29]. In another study in Western Cambodia, the most important risk factor for clinical *P. falciparum* episodes was living in a house where another clinical *P. falciparum* episode occurred (adjusted odds ratio (AOR):6.9; 95% CI 2.3–19.8) [36]. Sub-clinical infections of both *P. vivax* and *P. falciparum* were associated with clinical episodes of the same species (AOR: 5.8; 95% CI 1.5–19.7 for *P. falciparum* and AOR: 14.6; 95% CI 8.6–25.2 for *P. vivax*) and self-reported overnight visits to forested areas (AOR = 3.8; 95% CI 1.8–7.7 for *P. falciparum* and AOR = 2.9; 95% CI 1.7–4.8 for *P. vivax*) [36]. In the Amazon Basin of Brazil, subjects in index and neighbouring households were significantly more likely to be parasitaemic than control household members, after adjusting for potential confounders, and together harboured > 90% of the *P*. *vivax* biomass in study subjects.

Location was a more powerful predictor of finding malaria infections during case investigations than the demographics of the incident case. Larsen et al. found that various time-invariant measures of the environment, such as median enhanced vegetation index, the topographic position index, the convergence index, and the topographical wetness index, were all associated, as expected, with increased probability of finding a malaria infection during case investigations [37].

In Senegal, the adjusted relative risk (aRR) of infection was associated with residence in the index case household (aRR = 3.18, p < 0.05) and recent travel, including travel to Dakar (the capital city) (aRR = 19.93, p < 0.001), travel within the region (aRR = 9.57, p < 0.01), and to other regions in Senegal (aRR = 94.30, p < 0.001) [11]. Limiting blood testing to residents of the index case compound and neighbours with recent travel or fever would have identified 20/23 (87%) of the infections through testing of 1173 individuals. In Eswatini, proximity to the index case was associated with a dose-dependent increased infection risk (up to fourfold) using LAMP. Considering individual-, index case-, and other RACD-level factors, the simple approach of restricting RACD to a 200 m radius maximized yield and efficiency [10].

Simulations done along the Myanmar-Thailand border revealed that in approximately half of the screenings for falciparum and 10% for vivax infections it would have been impossible to detect any malaria cases regardless of the screening strategy as the screening would have occurred during times when there were no cases. When geographically linked cases were present in the simulation, RACD would have only been successful at detecting most malaria cases using larger screening radii (150-m radius and above). Beyond this radius, RACD does not perform better than random screening of an equal number of houses in the village. Screening within very small radii detects only a very small proportion of cases, but despite this low performance, it is better than random screening with the same sample size [38]. The authors conclude that RACD for clinical cases using RDTs has limited ability in halting transmission in regions of low and unstable transmission as transmission is linked to high spatial heterogeneity of cases, acquisition of malaria infections outside the village, as well as missing asymptomatic infections [38].

Gerardin et al. using mathematical modelling to assess the impact of transmission intensity and local inter-connectedness on reactive activities in malaria control/elimination in the Southern Province of Zambia [39], predicted that the success of elimination campaigns in both low- and high-transmission areas is strongly dependent on stemming the flow of imported infections, underscoring the need for regional-scale strategies capable of reducing transmission concurrently across many connected areas. In historically low-transmission areas, results indicated that treatment of clinical malaria should form the cornerstone of elimination operations, as most malaria infections in these areas are symptomatic, and onward transmission would be mitigated through health system strengthening; RACD had minimal impact in these settings. In historically high-transmission areas, vector control and case management are crucial for limiting outbreak size, and the asymptomatic reservoir must be addressed through RACD or mass drug campaigns. They recommended RACD only for settings where transmission has recently been reduced rather than in all low-transmission settings [39].

### Screening tests used in RACD

RDTs were the commonest test used, the others being microscopy, DNA based polymerase chain reaction (PCR) and loop-mediated isothermal amplification (LAMP). In this review, 10 studies used RDTs, 7 used PCR and 18 used microscopy. LAMP was not conducted in any of the countries as part of routine investigation; the three instances in which LAMP was used were only for research purposes (Table 2). The most widely used diagnostic technique in most of the countries is microscopy.

**Table 2:** Methods used for screening and case confirmation by different countries

	Rapid diagnostic tests	Microscopy	PCR	LAMP	Microsatellite (STRs) genotyping
China		X	X		
Cambodia	X	X			
Myanmar	X	X	X		
Viet Nam	X	X			
Eswatini	X	X		X	
Senegal	X				
Colombia		X	X		X
Indonesia		X		X	
Ethiopia		X	X		
Kenya		X	X		
Zambia	X	X			
Thailand	X	X	X		
Bhutan		X			
Malaysia		X			
Nepal	X	X			
Philippines		X			
Korea	X	X	X		
Solomon Islands		X			
Sri Lanka	X	X			

The detection limit of microscopy or RDTs is typically 100 parasites/microlitre and in low endemic settings, a high proportion of asymptomatic infections fall below this threshold [40, 41]. Outside of research settings, more sensitive detection methods, such as PCR are impractical due to high cost, sophisticated training and resources required, and log turnaround time (several hours). The loop-mediated isothermal amplification (LAMP) provides the sensitivity of PCR with fewer requirements, but its costs and cost effectiveness in RACD is unclear. However, testing is not at point-of-care and requires a laboratory, but the assay is simple, does not require sophisticated equipment and can be performed within a day [10, 42].

In Eswatini, compared to RDT, LAMP showed a threefold and 2.3-fold higher yield to detect infections (1.7% vs 0.6%) and hotspots (29.7% vs 12.7%), respectively. Hotspot detection improved with ≥ 80% target population coverage and response times within 7 days [10].

In a Namibian study, the sensitivities of RDTs and LAMP compared to nPCR were 9.30% and 95.50%, respectively, and specificities were 99.27 and 99.92%, respectively; LAMP carried out on collected RDTs had a sensitivity and specificity of 95.4% and 99.9% compared to nPCR carried out on drop blood spots (DBS). LAMP performed equally to nPCR for the identification of *P. falciparum* infections [43].

Among 2802 persons in Cambodia who were screened by PACD, 33 cases were detected by PCR (6 by RDT) (1.07%). Subsequent RACD activities among 273 persons yielded 3 *P. falciparum* cases (1.1%) by PCR (0 by RDT) [44].

In Zambia, testing by PCR revealed a *P.falciparum* gametocyte rate of 2.4% (2/87) among index case household members and 0% (0/141) among other contacts (p = 0.145) [9]. PCR identified four cases which were missed by routine microscopy in a study in Thailand [34]. Four rounds of microscopy-based RACD in the Amazon-basin of Brazil identified 49.5% of the infections diagnosed by qPCR, comprising 76.8% of the total parasite biomass circulating in the proximity of index households. Control households accounted for 27.6% of qPCR-positive samples; 92.6% of them were from asymptomatic carriers beyond the reach of RACD [33].

### Cost-effectiveness

Larson et al. summarizing a framework for evaluating the costs of malaria elimination interventions applied to RACD implemented through 173 health facilities across 10 districts in Southern Province of Zambia estimated that the mean annual cost per Health Facility Catchment Area was USD 1177 (median = USD 923; Inter Quartile Range USD 651–1417). Variation in costs was driven by the number of CHWs and passive cases detected. CHW-related costs and data review meetings accounted for the largest share of costs and RDTs and drugs accounted for less than 10% of the total costs [26].

In China, Indonesia and Thailand in which a monitoring and evaluation tool developed by Cotter et al. was piloted, the average monthly costs for conducting case investigation and RACD activities varied between the study areas (min USD 844.80–max USD 2038.00) for malaria personnel, commodities, services and other costs [31]. The authors surmised that the average monthly costs cannot be compared between countries due to different price structuring formulae and differences in the numbers screened.

### Challenges and limitations of RACD

Even though countries have successfully implemented various RACD techniques, there have been many challenges. A major challenge has been adhering to timelines. Achieving the three-day target in China was difficult due to logistic reasons such as sample collection, transportation and expedition by hospital staff to local CDC staff. Since PCR confirmation takes place in centralized laboratories, obtaining results and household investigations takes more than 3 days. Although household investigations should be done by local CDC’s staff, very often it is the hospital staff that does the investigations [22].

Reduced capacity due to limited diagnostic skills, shortage of primary healthcare staff and decreasing vigilance of malaria cases have been reported by field staff in China as a challenge for case reporting within 1 day. Lack of knowledge on malaria and its early presentation also hampers early detection. Even though RDTs are easier to perform, they are sometimes not available at primary healthcare centres. Despite a mobile-phone based short messaging alert system being in place to notify a case to the local Chinese Centre for Disease Control and Prevention [8], China’s web-based reporting system begins at township level and village clinics cannot fully participate in the surveillance system [45].

Challenges have been posed at case investigation level as well. Complexity of the procedures, difficulties in transportation, limited working time and other individual aspects may lead to delays in case confirmation. Old equipment coupled with limited experience of health workers hindered correct diagnosis and species identification. Case classification is done based on travel history alone [8] and is sometimes problematic due to incomplete travel histories. Even though, genotyping is helpful in distinguishing the geographical origin of the infection, standardized genotyping methods are not yet defined and unlikely to be completed within the three-day window [8]. Quality control of case investigations is difficult and sometimes accuracy and classifications are doubtful [45]. When PCR is used, confirmation of all networks to detect sub-microscopic infections within 7 days is difficult to achieve [8].

Foci investigation in 7 seven days is especially difficult during the transmission season when locally transmitted case numbers are highest and determination of foci is the most difficult [8], lack of human resources to make a professional judgement especially when a course of action needs to be determined due to differences of opinion and due to transportation issues in remote areas. Lack of a SOP often led to uncertainty in decision-making (for example, the radius to carry out RACD) [45]; sometimes, personnel who carried out RACD activities did not have a clear idea about the minimum geographic screening radius [31]. Along the China-Myanmar border, 65% of healthcare workers correctly stated that case investigation and RACD should occur in 3 and 7 days, respectively, 76% stated that all household members should be tested during RACD and 42% knew that it has to be conducted by visiting each household; knowledge of the minimum geographic radius to test around an index case household varied greatly [46].

Declining motivation for detection of cases and conducting investigations, increasing number of returning migrant workers from malaria-endemic countries, and the complexity of establishing functioning multi-sectoral collaboration teams have also been identified as challenges [45].

A pilot tool to evaluate RACD activities in Jiangsu province in China revealed that proper SOPs, organizational structures and documentation protocols for index cases and RACD were available in all healthcare facilities in the province [31]; 100% of RACD activities were completed during the specified time frame [31]. Implementation of the 1-3-7 strategy had varying success rates based on the area it is being implemented in.

A major limitation of the “1-3-7” strategy is when foci investigation are carried out by hospital doctors which may influence quality of data [22]. Another challenge was the difficulty in consolidating data on focus investigations and response for imported cases due to lack of clear implementation guidelines for county staff [22]. In China, IRS and RACD in potential active/active foci in foci investigation have been identified as challenges [45]. For RACD, standard operating procedures were lacking, the radius within which RACD should be conducted was not specified (ranging from 50–200 m) and community acceptance was poor. Active screening of migrant workers and their peers upon return to China was identified as a lesson learned. Furthermore, screening the workers’ social networks, potentially through information provided by export labour companies, facilitated the detection of potential cases [45].

In Kenya, a number of logistic challenges in conducting RACD have been highlighted as challenges [25]. The household location of each clinical case had to be recorded and communicated to teams ready to conduct follow-up activities. The identification of the size of the focus, and thus the number of households to target, required a detailed understanding of transmission epidemiology. The authors concluded that following-up index cases helps to identify asymptomatic cases but is unlikely to have a major impact on transmission in a hyper-endemic environment. They also highlighted the fact that given the logistic challenges to achieve high coverage of RACD, control programmes need to weigh the increased chance to detect secondary cases vs. activities targeting the whole community, which might be more cost effective [25].

Within the first year of implementation of a reactive screen-and-treat programme in Zambia, community health workers followed up 32% of eligible index cases; 66% of residents were at home in the index case households and 58% in neighbouring households [13]. Forty-one neighbourhood households of 26 index case households were screened, but only 13 (32%) were within the 140-m screening radius as specified in the country’s guidelines [13]. The authors conclude that with limited resources, coverage and diagnostic tools, reactive screen-and-treat will likely not be sufficient to achieve malaria elimination in this setting. They also surmise that high coverage with reactive focal drug administration could be efficient in decreasing the reservoir of infection and should be considered as an alternative strategy [13].

Although RDT is a quick and easy method to screen, often it misses detecting infections; PCR has shown to be better at detecting cases. Microscopy is also used in a majority of countries. However, lack of training and unavailability of equipment can be challenging when using this method. In Zambia, microscopy is limited to urban areas and referral hospitals. In rural health centers and at community level, the majority of the cases are confirmed by RDT [47]. In Myanmar, basic health staff and village health volunteers primarily use RDTs rather than microscopy for diagnosis of cases [48]. The primary and sole use of RDT for diagnosis may miss low-density malaria infections and elimination targets [22, 32, 45].

PCR is superior to microscopy in detecting malaria infections. PCR detected 2.2-fold higher *Plasmodium* infections as compared to microscopy [32]. In Kenya, PCR detected a higher number of cases than microscopy among members of the index case household, neighbours and persons living 500 m away from the index case [25].

Hustedt et al. found 0.5% positive cases by RDT and 1.1% using PCR during RACD. In the households assessed as a comparison group, 0 cases were identified by RDTs and 25 were identified by PCR [29].

Cambodia uses RDTs to identify infected persons. When RDTs were used to screen household members, only 1 case was found to be positive, whereas the number increased to 20 with PCR [44]. When the target population was expanded to include co-exposed individuals, RDT detected 6 cases whereas PCR detected 11. When screening was done in a wider target population using PCR, the overall positivity rate increased to 3.9% (31 out of 785) with the highest positivity rate reported among co-exposed. There was a significant association between the test used and the detection rate, with PCR having the higher detection rate (6.8% vs 3.2%, p = 0.03) [44]. Moreover, 75% of the samples from co-exposed individuals showed the same genotype as the sample from the index case.

In Colombia, the number of cases detected by PCR (93 cases) was significantly higher (5.6 times) than that detected by microscopy (16 cases) [27] strengthening the need to re-evaluate the diagnostic methods used in different types of epidemiological settings. Unavailability of resources is a major problem in accommodating these techniques in RACD approaches. In Ethiopia, detection by PCR is largely confined to PCR facilities and not currently used in the national malaria control programme [32].

Studies have shown that loop-mediated isothermal amplification (LAMP) is a better option that can be used to detect malaria infections. In an Indonesian study, one out of three who were detected positive for malaria by microscopy was false positive by LAMP. Of 1492 negative by microscopy, 5 were false negatives by LAMP. LAMP was more costly when compared to microscopy but more cost-effective for the detection of infections in scenarios with higher prevalence of infection using more sensitive diagnostics [49].

The confirmed presence of sub microscopic infections represents an important public health problem, as the unidentified positive cases will not receive treatment, and will maintain transmission [50]. RACD combined with parasite genotyping allows a better assessment of the transmission patterns [50].

RACD, though widely implemented [51], is operationally challenging requiring significant human resources, commodities, and time of an “on-call” team to conduct screenings in villages, often travelling long distances to reach remote locations [49]. There are also limitations with the standard diagnostics used, microscopy or RDTs, to detect low-density infections. Highly sensitive diagnostics are available but the costs and cost-effectiveness of using them is unclear [49].

There is little evidence available to support countries in deciding which methods to maintain, change or adopt for improved effectiveness and efficiency [4]. The development and use of common evaluation metrics for these activities will allow malaria programmes to assess performance and results of resource-intensive surveillance measures and, may benefit other countries that are considering implementing these activities [4]. There is very little information on how RACD programmes work in practice, if they achieve their goal, and if they are cost-effective, with little evidence to guide practice.

## Conclusions

Published literature on RACD, and case and foci investigations has mostly assessed the process of the activity. Most studies have documented that the yield of positives in RACD has been highest in the index case household and the immediate neighbourhood of the index case. LAMP and PCR, though more costly, have detected more cases than using only RDTs. Using PACD and RACD together have yielded better positivity rates than either one alone.

The guidelines for case and foci investigation, and RACD and PACD, are not universally adopted and are country specific. Some of the limitations and challenges identified include lack of proper guidelines, logistic issues and problems with public compliance. Developing generic guidelines for RACD will enable countries to adapt them accordingly for focussed implementation with the broader aim of malaria elimination.

None of the studies had compared the effectiveness or cost-effectiveness of RACD in elimination settings *vis*-*a*-*vis* no RACD. Although there is no documented evidence that RACD is useful in malaria elimination settings, most authors have opined that RACD is necessary for malaria elimination. It has been observed in Myanmar, that the yield of positives following case and foci investigations is very few in number. Given the limited geographic area covered in case investigations in most countries, it is unlikely to detect a significant number of infections in elimination settings. Even though evidence for RACD detecting infections during case and foci investigations is lacking to justify its continued application, just conducting it may have some effects on motivating staff and on keeping the malaria elimination drive on the radar; a qualitative study may be useful to discern this.

RACD is a strategy most countries are adopting to control or eliminate malaria. However, the success of RACD depends largely on the time taken to respond to case notifications, availability of resources and skill and knowledge of the personnel involved. China’s 1-3-7 strategy is a popular method and a number of countries have adopted it in their malaria control programmes. However, careful review should be done to adapt the method to suit the context and resources available. Lack of standard operating procedures on mediating the response is lacking in many settings and this is seen as a major hindrance in effective functioning of the RACD process. Lack of knowledge in the target populations, the target radius and how to carry out the RACD process is a major challenge in the decision-making process. Lack of resources for transportation and technology has been shown to slow communication and gaining access to settings where RACD should take place, a major reason why adhering to timelines has become a problem in many settings. Effective implementation of RACD requires skilled labour, resources and multi-sectoral collaboration. Many countries rely on RDT and microscopy for case detection, both of which can have a high possibility of missing cases, which can be a problem in a setting where elimination is the goal. RACD appears to be most effective when used in stable populations and performed including household members, co-exposed persons and others residing in a defined radius from the index case. It can be more effective when a detection method like PCR or LAMP is used for case detection. However, use of these techniques are costly and require expertise.

Given the available limited evidence, this report provides the following guidelines that countries may consider:

1. RACD is only applicable in elimination and prevention of re-establishment settings.
2. Detection of an imported case, a relapse, a recrudescence or an induced case in a currently or previously non-endemic area or during a non-transmission season does not warrant RACD.
3. RACD may be considered in currently or previously endemic areas in elimination and prevention of re-establishment settings, if
  - there is a suspicion or potential of transmission.
  - confined to a limited geographic area that includes household members and members of the immediate neighbourhood.
  - all necessary documentation of the process including SOPs have been developed and all staff carrying out activities are trained.
  - adequate logistic support including trained staff can be provided to conduct the time bound activities as planned.
  - the process can be closely monitored.

RACD will be affordable only if microscopy or RDTs are used. In elimination and prevention of re-establishment settings, RACD findings are important in case classification as the findings may provide evidence of the existence or absence of indigenous transmission. Whether to adopt RACD as an integral part of elimination or prevention of re-establishment strategies is an individual country decision based on its affordability, the health system, organisational structure and political commitment.

## Data Availability

Not applicable.

## Abbreviations

ACD: Active Case Detection
China CDC: Chinese Centre for Disease Control and Prevention
CHW: Community Health Worker
CISDCP: China Information System for Disease Control and Prevention
DNA: Deoxyribonucleic Acid
HFCA: Health Facility Catchment Area
LAMP: Loop-mediated Isothermal Amplification
PACD: Proactive Case Detection
PCD: Passive Case Detection
PCR: Polymerase Chain Reaction
RACD/RCD: Reactive Case Detection
RDT: Rapid Diagnostic Test
STR: Short Tandem Repeats
WHO: World Health Organization
